# Cryptolepine, the Main Alkaloid of the Antimalarial *Cryptolepis sanguinolenta* (Lindl.) Schlechter, Induces Malformations in Zebrafish Embryos

**DOI:** 10.1155/2019/7076986

**Published:** 2019-07-08

**Authors:** Kwesi Boadu Mensah, Charles Benneh, Arnold Donkor Forkuo, Charles Ansah

**Affiliations:** ^1^Department of Pharmacology, Faculty of Pharmacy and Pharmaceutical Sciences, Kwame Nkrumah University of Science and Technology, Kumasi, Ghana; ^2^Department of Pharmacology, School of Pharmacy, University of Health and Allied Sciences, Ho, Ghana

## Abstract

**Background:**

Previous studies on cryptolepine, the antimalarial and cytotoxic alkaloid of *Cryptolepis sanguinolenta*, showed that it preferentially accumulates in rapidly proliferating cells and melanin-containing tissues. Subsequently, we demonstrated that cryptolepine was toxic to murine embryos *in vivo* but no signs of teratogenicity. *in vivo* developmental studies can be confounded by maternal effects. Here, we hypothesized that cryptolepine-induced embryo toxicity occurs at least partly through direct inhibition of embryogenesis rather than indirectly through the induction of maternal toxicity.

**Aim:**

To determine the effects of cryptolepine on developing zebrafish embryos *ex vivo*.

**Methods:**

Healthy synchronized zebrafish eggs were treated with cryptolepine (10^−1^ − 5 × 10^2^ *μ*M), benzyl penicillin (6 − 6 × 10^2^ *μ*M), or mercury chloride (3.7 × 10^−1^ − 3.7 × 10^1^ nM) from 6 to 72 hours postfertilization. Developing embryos were assessed at 24, 48, 72, and 96 hours under microscope for lethality, hatching rate, and malformation.

**Results:**

LC_50_ for cryptolepine in the study was found to be 260 ± 0.174 *μ*M. Cryptolepine induced dose- and time-dependent mortality from the 24 to 96 hours postfertilization. Lower cryptolepine concentration (<100 *μ*M) caused mortality, approximately 15–18%, only after the 48 hours postfertilization. The most sensitive period of embryo lethality corresponded well with the pharyngula (24 to 48 hours) and hatching (48 to 72 hours) stages of embryonic development. Cryptolepine (10^−1^ − 5 × 10^2^ *μ*M) dose dependently inhibited the hatching rate. At doses above 500 *μ*M, hatching was completely inhibited. Mercury chloride (3.7 × 10^−1^ − 3.7 × 10^1^ nM), used as positive control, induced a consistent pattern of embryo lethality at all stages of development, whereas benzyl penicillin (6 − 6 × 10^2^ *μ*M), used as negative control, did not induce any significant embryo lethality. Morphological examination of (postfertilization day 5) of eleutheroembryos treated during embryonic development with cryptolepine showed decreased body length (growth inhibition), decreased eye diameter and bulginess, enlarged pericardia, and enlarged yolk sac and muscle malformations.

**Conclusion:**

Cryptolepine induces malformations, growth retardation, and mortalities in rapidly dividing zebrafish embryos *ex vivo*.

## 1. Background

**Figure 1:**
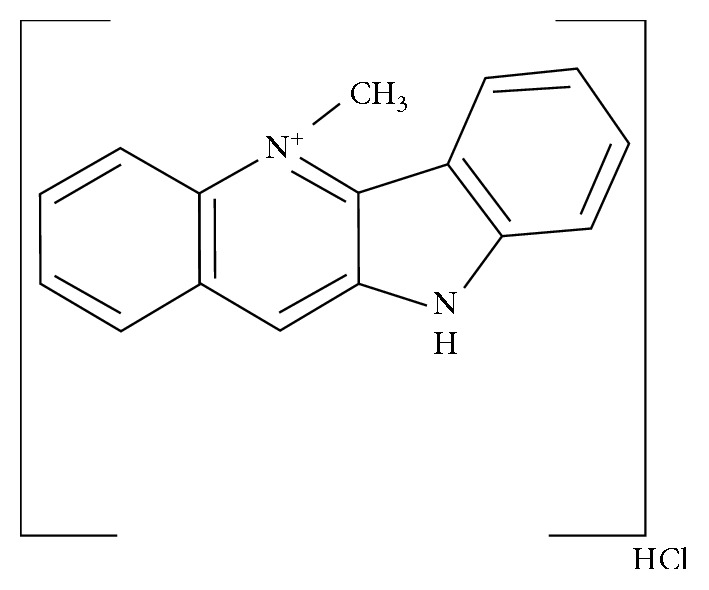
Cryptolepine hydrochloride (5H-quindoline, 5-methyl-, monohydrochloride; 5-methyl-5H-quindoline hydrochloride), the main indole alkaloid of the plant *Cryptolepis sanguinolenta* of family Periplocaceae.

Extracts of the West African antimalarial plant, *Cryptolepis sanguinolenta*, have been used in the management of infectious and noninfectious diseases in African Traditional Medicine for centuries [1]. Clinically, it has been proven to be unique in the management of malaria due to its rapid blood schizonticidal activity and potent anti-inflammatory activity [2–4]. Many biological and pharmacological effects of the plant extract have been attributed to the main alkaloid, cryptolepine (Figure 1); hence, cryptolepine is widely used for mechanistic studies for the antimalarial plant. Furthermore, cryptolepine demonstrates significant synergy with conventional antimalarial substances [5] and has shown good gametocidal activity against *Plasmodium falciparum* [6]. In view of this, cryptolepine and its analogues are under development as potential next-generation antimicrobial agents [7, 8]. Despite this potential, cryptolepine and the plant extract is cytotoxic to mammalian cells [9]. The mechanisms involve inhibition of the DNA supercoiling enzyme, topoisomerase II, and DNA intercalation [10–12]. Several studies indicate cryptolepine and its analogues may be a source of novel lead molecules for cancer chemotherapy [13, 14].

We had earlier demonstrated that aqueous extract of *Cryptolepis sanguinolenta* used as an antimalarial agent adversely affects mammalian reproduction [15]. It inhibited ovulation in lagomorphs and spermatogenesis in murine models [16, 17]. It was lethal to murine embryos and delayed postnatal growth and attainment of physical and developmental landmarks in mice [18]. There were no malformations or teratogenicity in the study. The mechanisms underlying these observations were not clearly delineated. It was, however, evident that the effects of cryptolepine on embryos may have been mediated partly through the induction of maternal stress and toxicity [18]. To be able to determine the effects of cryptolepine on embryonic development without the maternal influence, an *ex vivo* approach may be ideal.

The zebrafish, *Danio rerio* (Hamilton–Buchanan (1822)), has become a rapid, sensitive, and high-throughput model for the preclinical chemical screening tool [19, 20]. Unlike the traditional murine models, maintenance, husbandry, and reagent cost are low. It is very responsive to chemical insult and shares a genome of 85% homology with that of humans, making it a good model for predicting human effects [21, 22]. It is particularly suited for developmental toxicity studies because it produces large amounts of transparent eggs under simple laboratory conditions with rapid embryonic development. Distinct critical developmental stages constitute zebrafish embryogenesis [23]. Embryos remain transparent for unobstructed observations of morphology until the start of dense pigmentation 30–72 hours postfertilization. Using zebrafish embryos, the study aims to determine the effects of cryptolepine, the main alkaloid of *Cryptolepis sanguinolenta* on developing zebrafish embryos, Danio rerio, Hamilton–Buchanan (1822).

## 2. Materials and Methods

### 2.1. Isolation of Cryptolepine

Dried roots of *Cryptolepis sanguinolenta* were obtained from Center for Plant Medicine Research, Mampong, Akuapem, Ghana. It was authenticated at the Department of Pharmacognosy, KNUST, and compared to a voucher specimen KNUST/HM1/2008/L056 at the herbarium of the Department of Pharmacognosy and Herbal Medicine, Faculty of Pharmacy and Pharmaceutical Sciences, College of Health Sciences, KNUST, Kumasi, Ghana.

#### 2.1.1. Isolation and Purification of Cryptolepine

Methods of extraction, isolation, purification, and quantification of cryptolepine were as described in our earlier research [5, 6]. In brief, pulverized roots of dried *Cryptolepis* (900 g) were exhaustively extracted with methanol (3 L) by Soxhlet (50°C for 48 h) to give a dark crude alkaloid extract which was concentrated *in vacuo*. This was followed by a combination of liquid-liquid extraction and column chromatography. The isolated cryptolepine was identified by the ultraviolet-visible spectroscopy (peak wavelength of absorption was 223, 282, and 369 nm, purity of ∼99.5%) and melting point. Minor impurities include neocryptolepine and quindoline.

#### 2.1.2. Chemicals and Drugs

Crystalline benzyl penicillin (CAS RN: 69-57-8, purity 96.0–102.0% Troge Medical GmBH, Hamburg, Germany) and mercury chloride (CAS RN: 7487-94-7, purity 99% BDH, Poole, England) were used as control for the study. Agarose-G10 (Gene Co. Ltd., Spain) was used to immobilise eleutheroembryos.

#### 2.1.3. Animals

Adult zebrafish (*Danio rerio*, 12 weeks old, wild-type (WT) strain) were obtained from Aquarium Marshall Ltd. (Accra, Ghana). They were kept at the aquarium unit of Department of Pharmacology, Faculty of Pharmacy and Pharmaceutical Science, Kwame Nkrumah University of Science and Technology, for 7 days for acclimatization. Zebrafish were housed in glass tanks (∼21 L) at an average density of 1.5 fish per litre (female-to-male ratio was 1 : 1) containing filtered dechlorinated water maintained at 22–25°C and under a 14 h : 10 h light-dark cycle. Each tank had separate water inlet and outflow to minimize cross contamination. To mimic their natural habitat, each housing tank was filled with gravels to a height of about 2 cm, and a fresh water plant (*Cabomba aquatica*) was submerged in each tank.

#### 2.1.4. Egg Collection

Methods used were as described by McGrath et al. [24]. Matured adult zebrafish were fed twice daily with gold fish flakes (Tetra Spectrum Brands Pet, LLC, 3001 Commerce St., Blacksburg, USA). Spawning tanks were placed into the fish tanks the evening prior to egg collection. At onset of light, spawning tanks were removed and the eggs were transferred to a different container. The fertilised eggs were initially washed with distilled water to remove visible debris in the water containing the embryos. We subsequently washed and plated the embryos again in E3 media, which contains the salts to prevent any form of osmotic shock. After observing under a microscope, eggs with dead embryos, whitish appearances, and abnormal shapes and which were unfertilized were removed with the aid of a micropipette through selective aspiration [23–25]. Healthy synchronized eggs with an intact chorionic membrane were selected for the study.

### 2.2. Effects of Cryptolepine on Zebrafish Embryogenesis: Lethality and Hatch Rate

Healthy synchronized eggs approximately 2 hours postfertilization (hpf) were transferred to the 6-well cell culture plate (Coster 6 well plate 3527 Corning Incorporated Corning, New York, USA) (*n* = 7–10). Each well was assigned to a dose level of cryptolepine (10^−1^ − 5 × 10^2^ *μ*M) and treated to estimate the effect of cryptolepine on embryogenesis. Results were recorded from 6 to 72 hours postfertilization. Benzyl penicillin (6 − 6 × 10^2^ *μ*M) and mercury chloride (3.7 × 10^−1^ − 3.7 × 10^1^ nM) were used as controls. Distilled water was used as vehicle control. A fixed volume of 4 ml of each drug and/or vehicle solution was administered to well plate based on its assigned treatment. Drug solutions were continually replaced daily (static renewal) using a micropipette. The dead embryos were removed from each well plate using a micropipette and documented. The well plates were covered and kept in the dark throughout the study. Embryo lethality was assessed 24, 48, and 72 hours postfertilization. Hatch rate was assessed 96 hours postfertilization.

### 2.3. Effects of Cryptolepine on Zebrafish Eleutheroembryos: Microscopic Examination of Day 5 Postfertilization

On day 5 postfertilization, agarose gel was used to immobilize the drug-treated zebrafish embryos. Eleutheroembryos (*n* = 5) treated during embryogenesis with cryptolepine, mercury chloride, or benzyl penicillin during the embryonic stage were selected from well plates using a micropipette, placed on a depressed slide for examination under the light microscope (LEICA DM 750, at ×4 and ×10 magnification). Images of the head and the tail region were captured for each of the eleutheroembryos for morphological examination. A thorough examination of body length, body curvature, cranium and facial, and skin was done. Length of body and heart rate were quantitated with the aid of Image J software (NIH and LOCI).

### 2.4. Statistical Analysis

Results presented were either descriptive or quantitative. Quantitative results were analyzed with graph pad prism, version 6.0. Statistical analysis is by one-way ANOVA using the Dunnett multiple comparison test. *p* < 0.05 was considered statistically significant.

## 3. Results

### 3.1. Effect of Cryptolepine on Mortality of Zebrafish Embryos

**Figure 2:**
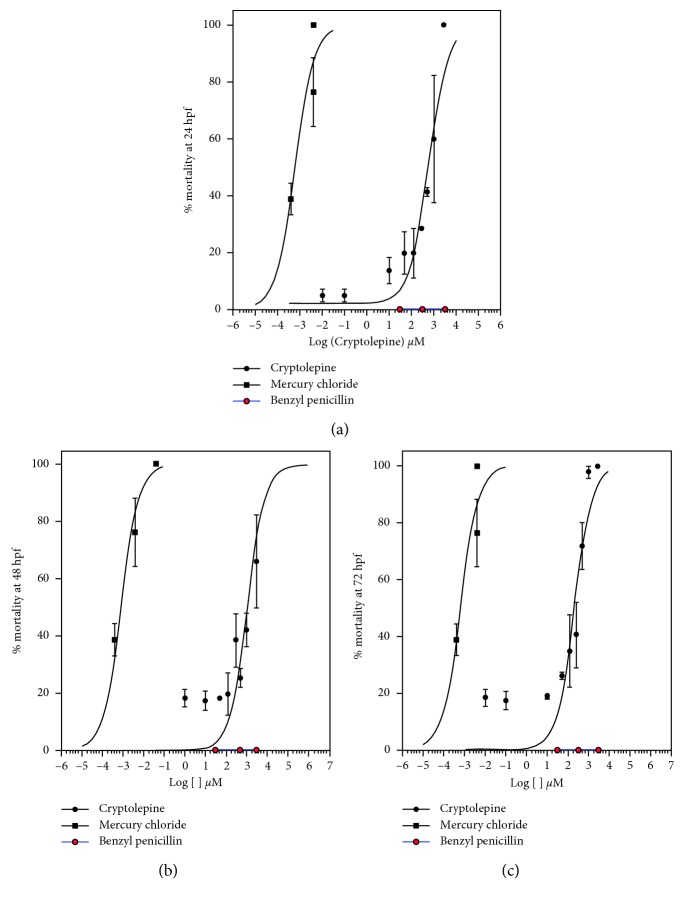
Effect of cryptolepine, mercury chloride, and benzyl penicillin on mortality of zebrafish embryos at (a) 24, (b) 48, and (c) 72 hours postfertilization.

Cryptolepine (10^−1^ − 5 × 10^2^ *μ*M) induced a dose- and time-dependent mortality in zebrafish embryos. Approximately 50% mortality occurred within 24 hours at doses of cryptolepine above 1 × 10^2^ *μ*M. This increased to over 90% within 72 hours of treatment (Figure 2). The most sensitive period of mortality corresponded with the pharyngula (24 to 48 hours) and the hatching (48 to 72 hours) period of zebrafish embryonic development. Lower cryptolepine concentrations (<1 × 10^2^ *μ*M) caused mortality only after prolonged treatment (>48 hours postfertilization). LC_50_ for cryptolepine in the study was found to be 260 ± 0.174 *μ*M at 24 hours of treatment. Benzyl penicillin used as negative control did not induce any significant embryo lethality throughout the duration of the study. However, mercury chloride induced significant embryo lethality at all the time points of the study. The shape, slope, and efficacy of the mercury chloride (concentration response curve) were not affected by the time of treatment (Figure 2). In the study, cryptolepine was less potent than mercury chloride.

### 3.2. Effect of Cryptolepine on Zebrafish Embryo Hatching Rate (96 Hours Postfertilization)

**Figure 3:**
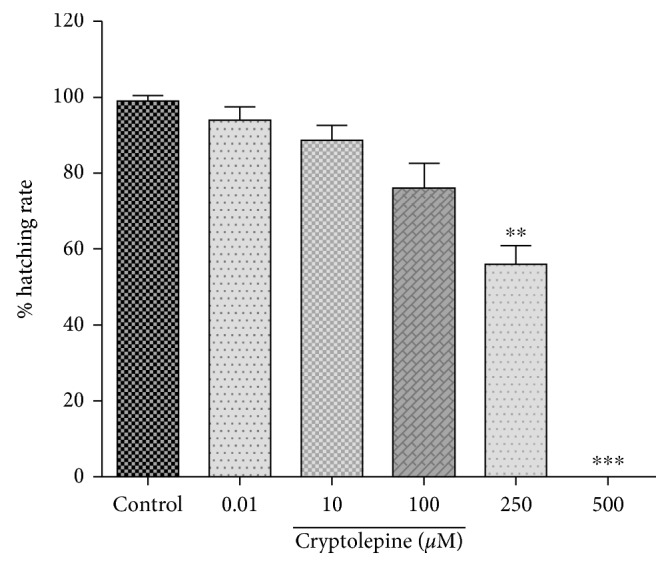
Effect of cryptolepine treatment during zebrafish embryogenesis on the hatching rate of 96 h postfertilization. Statistical analysis is by Dunnett's multiple comparison test. ^*∗*^*p* < 0.01; ^*∗∗∗*^*p* < 0.001.

Cryptolepine (10^−1^ − 5 × 10^2^ *μ*M) treatment decreased the hatching rate linearly with an increased dose (Figure 3). At doses of 250 *μ*M, approximately 40% failed to hatch and complete inhibition of hatching occurred at a dose around 500 *μ*M of cryptolepine (Figure 3). Benzyl penicillin (6 − 6 × 10^2^ *μ*M) did not affect the hatching rate. Methyl mercury (3.7 × 10^−1^ − 3.7 × 10^1^ nM) decreased the hatching rate with increased dosing significantly after 3 nM.

### 3.3. Effect of Cryptolepine on Zebrafish Eleutheroembryos' Developmental Landmarks

#### 3.3.1. Growth Retardation

**Figure 4:**
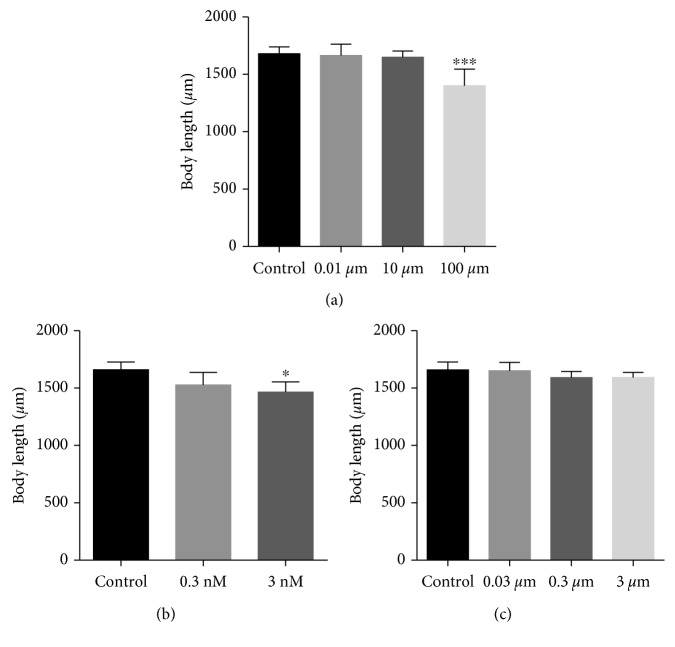
Effect of (a) cryptolepine, (b) mercury chloride, and (c) benzyl penicillin on body length of zebrafish eleutheroembryos. Statistical analysis is by Dunnett's multiple comparison test. ^*∗*^*p* < 0.05; ^*∗∗∗*^*p* < 0.001.

Cryptolepine inhibited growth in zebrafish eleutheroembryos pretreated from 2 to 72 hours postfertilization. Growth retardation was indicated by a reduction in overall body length and width. This was statistically significant at doses of cryptolepine above 1 × 10^2^ *μ*M (Figure 4). Cryptolepine induced growth retardation relative to controls quantitated to be 0.83%, 1.73%, and 16.6% at 10^−2^, 10, and 1 × 10^2^ *μ*M, respectively. Mercury chloride used as positive control also induced growth inhibition significantly at 3 nM. Percentage inhibitions induced by mercury chloride 7.9 and 11.63 at 0.3 and 3 nM, respectively. Benzyl penicillin did not affect growth of eleutheroembryos at the doses used during the study (Figure 4).

### 3.4. Cryptolepine Treatment on Zebrafish Morphology

#### 3.4.1. Craniofacial Examination

Zebrafish eleutheroembryo heads were profiled and viewed full face for malformations. The alignment of upper and lower jaws and the angle was examined. There were no differences between cryptolepine-treated eleutheroembryos and controls. Craniofacial notches, furrows, or distortions appeared identical.

#### 3.4.2. Eye Malformations

**Figure 5:**
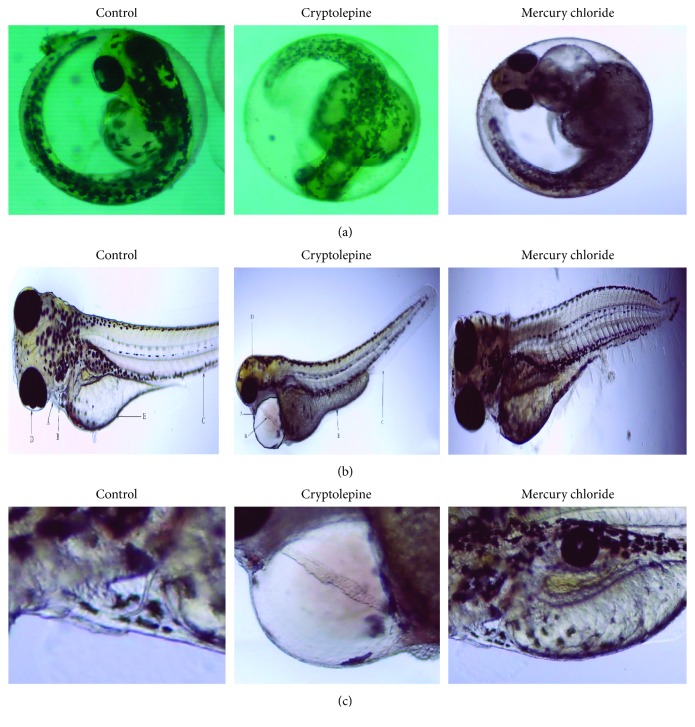
(a) Representative micrographs of overt toxicity seen in zebrafish embryos exposed to solvent control, cryptolepine 5 × 10^2^ *μ*M, and 3 × 10^−2^ *μ*M mercury chloride. The micrographs were taken at 72 hpf (×4). (b) Morphology of eleutheroembryos treated with cryptolepine and mercury chloride compared to the solvent control at postfertilization day 5. A = pericardial area; B = heart wall; C = somites; D = eye; E = yolk extension. (c) Photomicrograph of the pericardial area of zebrafish eleutheroembryos treated with cryptolepine and mercury chloride compared to the solvent control at postfertilization day 5.

Zebrafish eleutheroembryos exposed to cryptolepine, mercury chloride, or benzyl penicillin during embryogenesis were examined for bulginess, size, symmetry, and position. Cryptolepine-treated zebrafish eleutheroembryos had a small eye diameter. The eye appeared sunken instead of being bulged as seen in other groups. There was no observable difference in eye pigmentation (Figure 5(b)).

#### 3.4.3. Skin and Body Curvature Examinations

The skin of zebrafish was checked carefully for continuity, abnormalities, pigmentation, tone, and texture. There was no notable difference between cryptolepine-treated eleutheroembryos and controls. Cryptolepine-treated animals did not show abnormal swellings or subdermal hematomas (ecchymosis). Overall body curvature was similar amongst all groups.

#### 3.4.4. Yolk Sac Extension and Muscle Malformation

Treatment of zebrafish embryos with cryptolepine resulted in yolk sac edema and signs of muscular atrophy in the eleutheroembryos. This occurred at 1 × 10^2^ *μ*M (Figure 5(b)).

#### 3.4.5. Pericardial Edema and Altered Heart Rate

Treatment of zebrafish embryos with cryptolepine increased the pericardial area of the zebrafish eleutheroembryos. Neither mercury chloride nor benzyl penicillin used as control affected the pericardial area of eleutheroembryos. Preliminary quantification showed that the effect was significant at 1 × 10^2^ *μ*M and the enlargement in the pericardial area corresponded with a decrease in the heart rate (Figure 5(c)).

## 4. Discussion

Zebrafish (*Danio rerio*, Hamilton–Buchanan (1822)) is widely gaining a reputation as a reliable scientific model for rapid screening of pharmaceuticals [26, 27]. It is very responsive to chemical insult and shares a genome of 85% homology with that of humans, making it a good model for predicting human effects [21, 22]. The popular antimalarial plant *Cryptolepis sanguinolenta* is cytotoxic and adversely affects reproduction and embryonic development in mammals [9, 13, 15]. Cryptolepine, the main indole alkaloid of the plant, accumulates preferentially in rapidly dividing cells of treated organisms [28]. Reported embryonic effects include lethality, intrauterine growth inhibition, and functional toxicities [15]. No embryonic and fetal malformations had been reported in any previous studies [15–18]. However, there were indications that maternal effects may have confounded developmental toxicity outcomes. This study therefore examined the effects of cryptolepine on zebrafish embryos [18].

In the study, cryptolepine induced significant embryonic death within 24 hours postfertilization. This observation in zebrafish embryos corroborate well with reports by other authors working using A549 cells, HEP G2, hepatome cell line, V79, and a Chinese hamster fibroblast cell line [29–31]. In those cited studies, the effective concentrations of cryptolepine were from 0.5 to 5.0 *μ*M. These doses induced significant cell cycle arrest with upregulation of proapoptotic gene p53 within 6 to 24 hours. In zebrafish, doses below 10^2^ *μ*M induced growth retardation, but embryos survive the effects of cryptolepine until 48 hours of treatment where significant mortalities appeared. The differences between the doses of the cell lines in previous studies and that of zebrafish may be due to the rapid development and increase in cell numbers and possible differences in pharmacokinetics cryptolepine rather than differences in sensitivity to effects of cryptolepine.

In the murine model, cryptolepine causes significant pregnancy loss from conception through embryogenesis, indicative of a possible contraceptive effect. The embryonic death may be secondary to extensive apoptosis induced by cryptolepine in the rapidly dividing cells. Cryptolepine has demonstrated pleiotropic mechanisms of inducing apoptosis in the cell cycle [29–31]. In whole animal studies, dead embryonic tissues subsequently undergo tissue resorption by the maternal system [32]. Evidence from this study shows that the contraceptive activity of cryptolepine is at least partly due to direct effects on the embryo rather than an alteration in maternal endocrine function or endometrial changes [15, 17].

The most sensitive period for embryonic susceptibility to cryptolepine was the pharyngula and hatching peroid. During the pharyngula period, there is development of the nervous system and the maturation of the brain to 5 distinct lobes occurs. Interestingly, mice exposed prenatally to develop a pattern of functional toxicity which could be explained by altered neurogenesis and brain development. Practically, functional toxicity is a manifestation of neurotoxicity. This study in zebrafish gives a better insight into the possibility of cryptolepine affecting brain and neuronal development. Furthermore, prenatal treatment of mice in our previous study with *Cryptolepis* caused a delay in eye opening on postnatal day 8 [18]. A delay in eye opening is indicative of alteration in neurogenesis. In this study, cryptolepine cause malformations in the eye of characterized by decreased diameter and reduced bulginess. The eye may be more susceptible to the effects of cryptolepine compared to other organs because of the affinity of cryptolepine for melanin [28]. This affinity preferentially favours accumulation of cryptolepine in the eye which may inhibit cell proliferation.

This study noted cardiac and muscular changes in fish exposed to cryptolepine. There was a decrease in the heart rate amongst animals treated with cryptolepine. The effect of cryptolepine on the heart rate showed that there was a significant decrease in the heart rate of zebrafish embryos treated with high concentration of cryptolepine (>100 *μ*M). There was also an increase in the pericardial area of zebrafish eleutheroembryos whose embryos were treated with high concentrations of cryptolepine. The mechanism underlying this effect is not known. In our previous study, we showed that mice treated prenatally with the aqueous extract of *Cryptolepis* showed altered sensorimotor activity and performed poorly in spontaneous locomotor and motor coordination studies [18]. It is possible that there could be a linkage between the cardiac and the muscular system and the performance on the rotarod experiment because of it effects on early embryonic tissues.

## 5. Conclusion

In conclusion, cryptolepine, the main alkaloid of the antimalarial *Cryptolepis sanguinolenta*, is embryotoxic to zebrafish. It induced embryo lethality, growth inhibition, malformation of the eye, and elongation of the pericardial area and the yolk.

## Data Availability

The data used to support the findings of this study are included within the article.
